# De Novo Osteogenic Sarcoma of Mastoid Bone

**DOI:** 10.1080/1357714021000022186

**Published:** 2002-06

**Authors:** Abdurrahman Işikdogan, Abdullah Büyükçelik, Selim Erekul, Ali Pamir

**Affiliations:** 1 Department of Medical Oncology Ankara University Faculty of Medicine Ibni Sina Hospital, Sihhiye Ankara 06100 Turkey; 2 Department of Pathology Ankara University Faculty of Medicine Ibni Sina Hospital, Sihhiye Ankara 06100 Turkey

## Abstract

The most common primary malignant tumor of the bone is osteosarcoma. Primary involvement of the craniofacial bones in osteosarcoma is relatively rare. The mandible and the maxillae are the most commonly affected bones of the head. Here, we report a rare case of de novo high-grade osteogenic sarcoma of the mastoid region of the temporal bone and discuss the diagnostic and therapeutic properties.


*Sarcoma* (2002) **6**, 79–81

Taylor & Francis healthsciences

CASE REPORT

### De novo osteogenic sarcoma of mastoid bone

ABDURRAHMAN IŞIKDOGAN[1], ABDULLAH BÜYÜKÇELIK[1], SELIM EREKUL[2] & ALI PAMIR[1]

*[1]Department of Medical Oncology, [2]Department of Pathology, Ankara University, Faculty of Medicine, Ibni Sina Hospital, 06100, Sihhiye, Ankara, Turkey*

**Abstract**
The most common primary malignant tumor of the bone is osteosarcoma. Primary involvement of the craniofacial bones in osteosarcoma is relatively rare. The mandible and the maxillae are the most commonly affected bones of the head. Here, we report a rare case of de novo high-grade osteogenic sarcoma of the mastoid region of the temporal bone and discuss the diagnostic and therapeutic properties.

**Key words:** *osteosarcoma, mastoid bone*

### Introduction

Osteosarcomas are the most common primary malignant tumors of the bone. The appendicular skeleton, in particular the long bones of the limbs, especially the distal end of the femur and the proximal end of the tibia are the most common primary sites. Osteosarcomas of the craniofacial bones are relatively rare and represent less than 10% of all osteosarcomas. The most commonly affected bones of the head are the mandible, followed by the maxilla. Osteosarcomas of extragnathic craniofacial bones are very rare, constituting fewer than 2% of all osteosarcomas.[1,2] We report a rare case of de novo osteogenic sarcoma of the mastoid region of the temporal bone.

### Case report

A 20-year-old man was admitted to a private medical center in May 2001, with a history of extensive painful swelling over the left temporo-mastoid region. He was also suffering from hearing loss in the left ear. Computed tomography (CT) scan of the mastoid showed a radio-dense mass with soft tissue density areas which caused destruction of mastoid bone, was extending to the external auditory canal. In addition, there was a bony fragmentation in the lesion, and loss of aeration in the mastoid bone (Fig. 1a). The tumor was excised totally in the hospital. Histopathological diagnosis was osteogenic sarcoma of the mastoid bone (Fig. 2). He was referred to the Department of Medical Oncology of Ybni Sina Hospital, Ankara University, Faculty of Medicine, in June 2001 for further treatment. The patient had none of the predisposing factors, such as Paget's disease of the bone, radiotherapy or fibrous dysplasia. There was no abnormality on general physical examination except a left postauricular incision scar. Hematological and biochemical parameters, including serum alkaline phosphatase, were normal. Postoperative CT scan showed the cavitation due to surgical removal and there was no sign of macroscopic residual tumor (Fig. 1b). No metastatic lesions were noted on chest CT and bone scintigraphy. The histopathological slides were revised and the diagnosis of high-grade medullary osteosarcoma was confirmed (Fig. 2). The combination chemotherapy regimen that we use for conventional osteosarcoma, consisting of ifosfamide, adriamycin, high-dose methotrexate, and cisplatin, was applied as adjuvant treatment. Adjuvant chemotherapy was completed and the patient has been alive for 11 months without any recurrence of the disease.

### Discussion

We present an aggressive medullary osteosarcoma arising from an unusual site, the mastoid region of

Correspondence to: Abdullah Büyükçelik, MD, Department of Medical Oncology, Ybni Sina Hospital, Faculty of Medicine, Ankara University, Syhhiye, Ankara 06100, Turkey. Tel.: +90–312–310–3333, Ext. 2132 & 2644; Fax: +90–312–312–1650; GSM: +90–535–894–9503; E-mail: drabdullah@superposta.com

ISSN 1357–714X print/ISSN 1369–1643 online/02/0200079–03 © Taylor & Francis Ltd
DOI: 10.1080/1357714021000022186

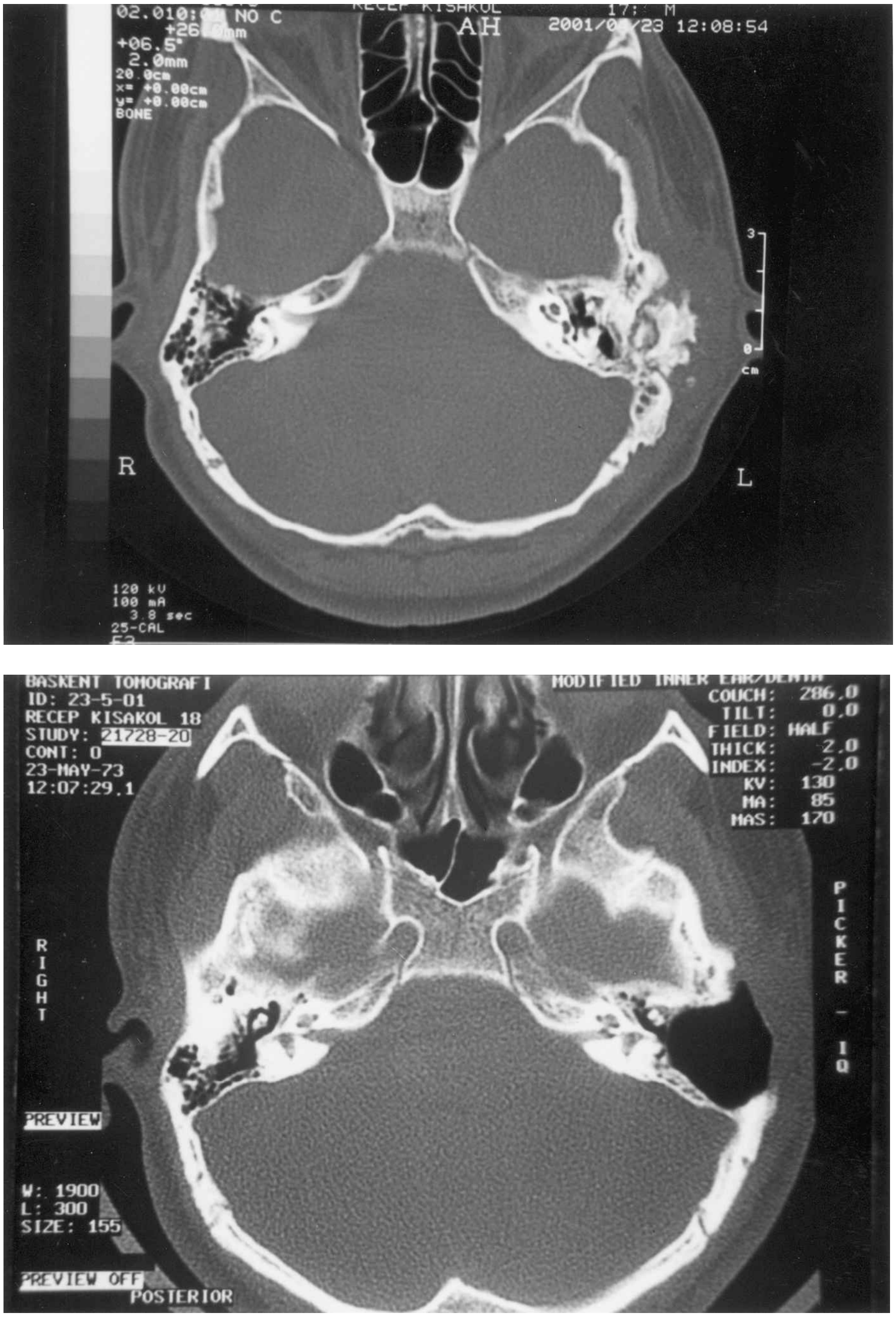

**Fig. 1.** *Computed tomography of temporal bones showed a mass of soft tissue that contained bone fragments, obliterating external auditory meatus and subcutaneous fatty tissue: destruction of mastoid bone and the loss of aeration in mastoid cells on the left side (a) and the cavitation in mastoidal bone after surgery (b).*

the temporal bone, developed in a patient without any predisposing condition.

Tumors of the mastoidal bone are uncommon, with the exception of osteomas.[1] Primary malignant tumors of this bone are very rare. Osteogenic sarcoma, which is the most common primary bone tumor, rarely occurs in the facial and the cranial bones. It may be located in the mandible and maxillae, but it is very rare in the temporal bone.[2–4] Osteosarcomas of extragnathic craniofacial bones constitute fewer than 2% of all osteosarcomas, and less than 0.5% of all primary bone tumors. These osteosarcomas may be 'classic' aggressive medullary type or parosteal osteogenic sarcoma, which is a low-grade surface bone tumor with a better prognosis than medullary osteogenic sarcoma.[5,6]

Nora *et al.* reported a series of 21 patients with osteosarcomas of extragnathic craniofacial bones.[2] They revised the archives of Mayo Clinic containing nearly 7000 bone tumors, including 1000 osteosarcomas, and only 21 cases affecting extragnathic craniofacial bones were found. These tumors were located over the entire calvarium The usual involved site was the occipital bone. Only one of these cases was located at the temporo-sphenoid region. Salvatti *et al.* also presented 19 cases of osteosarcoma of the skull,[7] only one of which was located at the temporo-parietal bone region. Sharma *et al.* reported a case of

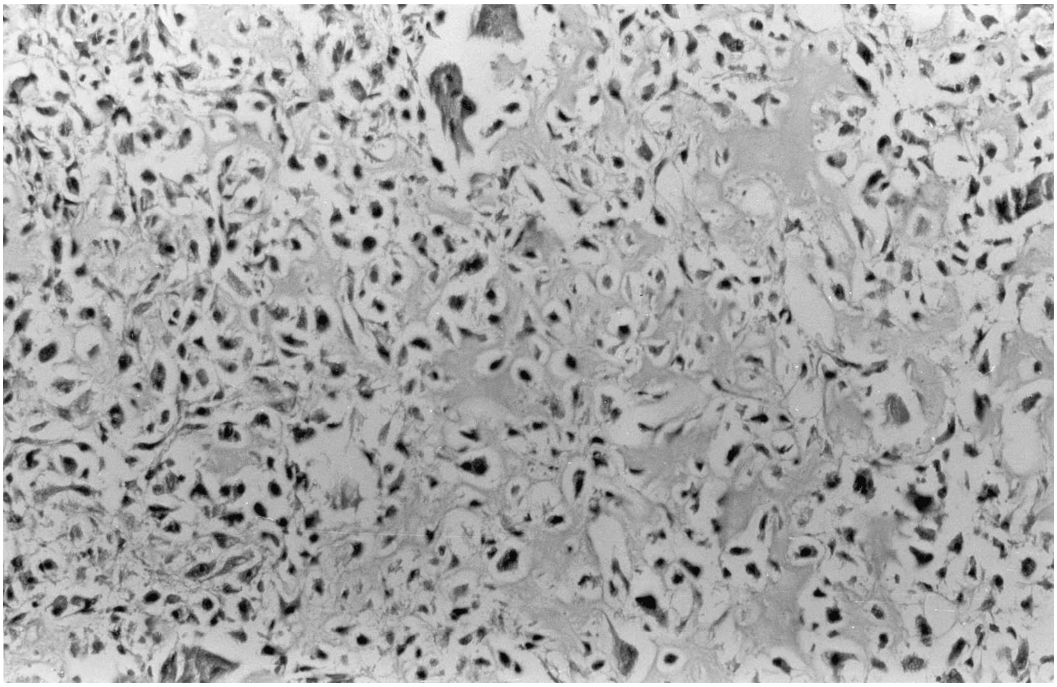

**Fig. 2.** *Histopathological examination of the mass in mastoid bone demonstrated many eosinophilic lace-like osteoid accumulations with atypical osteoblasts surrounding them HE (200x).*

classic osteogenic sarcoma of the temporal bone.[4] Seely and Gates[5] reported two cases of parosteal osteosarcoma arising from mastoid bone, and Kumar *et al.*[6] also reported eight patients with parosteal osteosarcoma of cranial bones, only two of them were arising from the mastoid region.

The skull is a more common site for secondary osteosarcomas. Paget's disease and prior radiotherapy are the most common predisposing conditions. The rate of the secondary osteosarcoma in patients of Nora *et al.*[2] and Salvati *et al.*[7] were reported as six of 21 (28%) and seven of 19 (36.8%), respectively. In these two series, the authors had not classified the cases as aggressive medullary or parosteal osteosarcomas, but the vast majority of these tumors were histologically described as being highly malignant.

Parosteal osteosarcomas with typical radiological appearance of sessile, densely ossified surface growth with radiating bone spicules that blend with surrounding soft tissue are usually low grade lesions and *en bloc* resection is curative in most cases.[5,6] Medullary high-grade osteosarcoma of the craniofacial region carries increased risk of local recurrences and distant metastases.[8] There is no reason to manage these patients in a different way from conventional osteosarcomas. They should be treated in a multidisciplinary fashion, with preoperative chemotherapy followed by radical resection and adjuvant treatment, similar to the successful treatment of conventional extremity osteosarcomas. Some limited experiences have already appeared in the literature.[7,9,10] The difficulty of radical resections in this region and limited effectiveness of radiotherapy in osteosarcoma may increase the importance of effective neoadjuvant and adjuvant chemotherapy.

### References

1. Malawer MM, Link MP, Donaldson SS. Sarcomas of bone. In: DeVita VT Jr, Hellman S, Rosenberg SA, eds. *Cancer*. 5th edn. Philadelphia, PA: Lipincott-Raven, 1997: 1789–852.
2. Nora FE, Unni KK, Pritchard DJ, *et al.* Osteosarcoma of extragnathic craniofacial bones. *Mayo Clin Proc* 1983; 58: 268–72.
3. De SK, Dey DD. Tumours of the mastoid temporal bone: with interesting cases in the paediatric age group. *J Laryngol Otol* 1988; 102: 582–7.
4. Sharma SC, Handa KK, Panda N, *et al.* Osteogenic Sarcoma of the temporal bone. *Am J Otolaryngol* 1997; 18: 220–3.
5. Seely DR, Gates GA. Parosteal osteogenic sarcoma of the mastoid bone. *Ann Otol Rhinol Laryngol* 1997; 106: 729–32.
6. Kumar R, Moser RP Jr, Madewell JE, *et al.* Parosteal osteogenic sarcoma arising in cranial bones: clinical and radiologic features in eight patients. *AJR Am J Roentgenol* 1990; 155: 113–7.
7. Salvati M, Ciappetta P, Raco A. Osteosarcomas of the skull. Clinical remarks on 19 cases. *Cancer* 1993; 71: 2210–6.
8. Rosen G, Forscher C, Mankyn HJ, Selch MT. Bone tumors. In: Bast RC Jr, Kufe DW, Pollock RE, Weichselbaum RR, Holland JF, Frei E III, eds. *Cancer medicine*. 5th edn. Hamilton, London: BC Decker, 2000: 1870–902.
9. Smeele LE, Kostense PJ, van der Waal I, Snow GB. Effect of chemotherapy on survival of craniofacial osteosarcoma: a systematic review of 201 patients. *J Clin Oncol* 1997; 15: 363–7.
10. Sunderasan N, Huvos AG, Rosen G, Galicich JH. Combined-modality treatment of osteogenic sarcoma of the skull. *J Neurosurg* 1985; 63(4): 562–7.

## References

[PDF_00166] De S. K., Dey D. D. (1988). Tumours of the mastoid temporal bone: with interesting cases in the paediatric age group.. J Laryngol Otol.

[PDF_00175] Kumar R., Moser R. P., Madewell J. E., Edeiken J. (1990). Parosteal osteogenic sarcoma arising in cranial bones: clinical and radiologic features in eight patients.. AJR Am J Roentgenol.

[PDF_00163] Nora F. E., Unni K. K., Pritchard D. J., Dahlin D. C. (1983). Osteosarcoma of extragnathic craniofacial bones.. Mayo Clin Proc.

[PDF_00179] Salvati M., Ciappetta P., Raco A. (1993). Osteosarcomas of the skull. Clinical remarks on 19 cases.. Cancer.

[PDF_00172] Seely D. R., Gates G. A. (1997). Parosteal osteogenic sarcoma of the mastoid bone.. Ann Otol Rhinol Laryngol.

[PDF_00169] Sharma S. C., Handa K. K., Panda N., Banerjee A. K., Mann S. B. (1997). Osteogenic sarcoma of the temporal bone.. Am J Otolaryngol.

[PDF_00187] Smeele L. E., Kostense P. J., van der Waal I., Snow G. B. (1997). Effect of chemotherapy on survival of craniofacial osteosarcoma: a systematic review of 201 patients.. J Clin Oncol.

[PDF_00191] Sundaresan N., Huvos A. G., Rosen G., Galicich J. H. (1985). Combined-modality treatment of osteogenic sarcoma of the skull.. J Neurosurg.

